# Toughening Mechanism and Nonlinear Water-to-Binder Ratio Effects in Multi-Scale Steel Fiber–Calcium Carbonate Whisker Reinforced Concrete

**DOI:** 10.3390/ma19194044

**Published:** 2026-09-22

**Authors:** Jiyang Wang, Xianmo Xu, Xiaoyao Ren, Lingbo Wang, Xiaohua Ji, Bocheng Xie, Pinghuai Zhou

**Affiliations:** 1 Institute of Advanced Engineering Structures, Zhejiang University, Hangzhou 310058, China; 22412186@zju.edu.cn (X.X.); 3220104859@zuaa.zju.edu.cn (X.R.); jixiaohua@zju.edu.cn (X.J.); 22512158@zju.edu.cn (B.X.); 2 Zhejiang Communications Construction Group Co., Ltd., Hangzhou 310007, China; wlingbo@zjjtgc.com; 3 Zhejiang Province Institute of Architectural Design and Research, Hangzhou 310006, China; anji18@126.com

**Keywords:** steel fiber, calcium carbonate whisker, multiscale reinforcement, water-to-binder ratio, toughening mechanism, orthogonal experiment

## Abstract

The toughening efficiency of multiscale fibers is strongly coupled with the matrix state, yet how the water-to-binder (w/b) ratio shifts the governing reinforcement mechanism remains unclear. Two w/b regimes representing distinct matrix conditions were therefore incorporated into a three-factor, four-level orthogonal design, with water, steel-fiber, and calcium carbonate whisker dosages as variables. Compressive, flexural, and direct tensile strengths, together with failure characteristics, were evaluated to track the transition in mechanical response across the two regimes. In the low w/b ratio regime, the w/b ratio controlled matrix compactness and interfacial quality, significantly affecting compressive and flexural strengths (*p* = 0.0005 and *p* = 0.0016, respectively). As the matrix became more susceptible to cracking, the contribution of w/b ratio diminished, whereas steel-fiber bridging became dominant and significantly influenced flexural strength (*p* = 0.0027). This regime transition demonstrates that the governing mechanism evolves nonlinearly from matrix-controlled strength to fiber-controlled crack resistance. Calcium carbonate whiskers delayed damage initiation through microfilling, crack-tip blunting, and microcrack bridging, but excessive dosages promoted agglomeration and local defects. Steel fibers subsequently restrained macrocrack growth through interfacial bonding, bridging, slip, and pull-out. Together, these actions established a hierarchical toughening mechanism spanning microdefect regulation and macrocrack bridging, transforming abrupt brittle fracture into progressive cracking while sustaining structural integrity. These findings clarify the matrix-dependent nature of multiscale reinforcement and provide a mechanistic basis for regime-specific cementitious composite design through coordinated optimization of matrix quality, microscale crack control, and macroscale bridging.

## 1. Introduction

Quasi-brittle fracture, localized macro-cracking, and sudden post-peak load degradation during service remain critical engineering challenges for concrete structures. Transitioning from brittle failure to a ductile, energy-dissipating fracture mode—often described as “cracking without rupture”—requires tailoring the microstructure to sustain load-carrying capacity throughout the post-peak softening phase [1,2,3,4]. Incorporating steel fibers (SFs) effectively mitigates catastrophic failure by transferring stress across crack flanks via bridging and pull-out mechanisms [5,6,7,8]. However, SF reinforcement efficiency depends on matrix density, interfacial bond strength, and fiber dispersion [5,7]. Because variations in the water-to-binder (w/b) ratio fundamentally govern the microstructural evolution of the interfacial transition zone (ITZ), the fiber bridging-pullout response exhibits pronounced stage-dependent, nonlinear behavior across different matrix strengths [9,10,11,12]. Consequently, evaluating SF toughening based solely on fiber volume dosage or geometry overlooks how the w/b ratio shifts the dominant toughening mechanism, hindering mechanism-based mix design.

At the microscale, calcium carbonate whiskers (CCWs) and microcrystalline inorganic fillers offer substantial toughening potential through pore refinement, heterogeneous hydrate nucleation, and microcrack deflection [13,14,15,16,17,18]. These micro-reinforcements densify the matrix paste and strengthen the ITZ, thereby elevating the macro-crack initiation threshold. Nevertheless, excessive whisker dosages trigger agglomeration and introduce initial defect clusters, offsetting densification gains and accelerating micro-crack coalescence [15,16,17,18]. This dual effect yields a non-monotonic toughening response governed by whisker dispersion, pore evolution, and the w/b ratio [13,17]. Although SFs and CCWs separately enhance macroscopic ductility and microcrack resistance, a unified explanation of how they synergistically regulate the entire fracture process across varying w/b ratios remains lacking.

Conventional fracture characterization relies on flexural load–displacement responses, total dissipated energy, and equivalent toughness indices [19,20]. While these parameters reveal phenomenological trends in ductile failure, they do not capture the underlying interfacial damage and microstructural evolution [21,22]. From a fracture mechanics perspective, w/b ratio-induced variations in ITZ porosity and fracture toughness alter stress intensity fields and damage zone sizes at the crack tip, thereby modulating the effective energy dissipation domain contributed by fiber bridging [23,24,25]. Incorporating the w/b ratio as an active design variable for multiscale reinforced composites is therefore essential to bridge the fragmented understanding of hybrid toughening.

To address these limitations, this study investigates how the w/b ratio affects the cracking and toughening process in SF–CCW composites. Specifically, we examine three key aspects: (i) how matrix densification influences SF bridging and pullout behavior [6,9,26,27,28,29]; (ii) the balance between CCW micro-reinforcement and agglomeration-induced defects [17,18,30]; and (iii) how these combined mechanisms can help guide mix design beyond simple trial and error [31,32]. We hypothesize that at low w/b ratios, a dense matrix and reinforced ITZ shift early-stage crack control toward microscale matrix densification, fully activating CCWs’ nucleation and pore-filling capacity. Conversely, at elevated w/b ratios, ITZ softening and matrix defect accumulation diminish crack-tip restraint, causing SF bridging to dominate post-peak energy dissipation [33,34]. Simultaneously, CCWs exert a non-linear effect: optimal dosage delays macro-crack formation via micro-bridging, whereas excessive additions introduce flaw sites that compromise softening efficiency [13,14,17,18].

Herein, multiscale SF–CCW-reinforced concretes across a systematic range of w/b ratios were formulated via close-packing optimization and orthogonal design. Through a comprehensive mechanical evaluation comprising compressive, flexural, and direct tensile tests, this study reveals how the matrix governs the synergistic reinforcement between micro-whiskers and macro-fibers under diverse stress states. By elucidating the transition of multiscale cracking behaviors across varying w/b ratios, these findings bridge the gap between empirical proportioning and multiscale failure mechanisms, providing a mechanistic foundation for tailoring high-performance cementitious composites.

## 2. Materials and Experimental Methods

### 2.1. Raw Materials

To construct a dense, defect-minimized cementitious matrix capable of accommodating multiscale crack-bridging mechanics, a ternary binder system was synthesized using P II 52.5, Portland cement (Tongling Conch Cement Co., Ltd., Tongling, Anhui, China), Grade I fly ash (Jianbi Power Plant Zhenjiang, Jiangsu, China), and undensified silica fume (Aken International Trading Co., Ltd., Shanghai, China). Micron-sized quartz powder (SiO_2_) was incorporated to further tailor the particle packing density and optimize the ITZ via synergistic physical filling and pozzolanic reactivity. The aggregate framework comprised 40/70-mesh calcined quartz sand (Tongliao Hongyuan Silica Sand Co., Ltd., Tongliao, Inner Mongolia, China) and 5–10 mm crushed basaltic stone (coarse aggregate), yielding a stable, low-void structural skeleton. Reference [35] presents the detailed chemical compositions of all raw materials.

A hierarchical two-phase reinforcement architecture was tailored to capture crack propagation across micro- to macro-length scales. At the micro-scale, acicular CCWs (mean diameter d = 8 μm, pH = 6.5, density: 2.69 g/cm^3^) (Jinan Quanxin Chemical Co., Ltd., Jining, Shandong, China) were deployed to promote localized nucleation (Figure 1a), refine C-S-H gel porosity, and arrest early-stage microcrack initiation. At the macro-scale, high-strength copper-coated SFs (length *l* = 12.8 mm, diameter *d* = 0.2 mm, aspect ratio *l*/*d* = 64, tensile strength *σ*_t_ = 2988 MPa) (Zhejiang Boen Metal Products Co., Ltd., Huzhou, Zhejiang, China) were integrated to govern post-cracking energy dissipation via robust crack-bridging stresses (Figure 1b). The physical parameters of the CCW and the macro-scale physical properties of the steel fibers are detailed in Table 1 and Table 2, respectively. To ensure uniform fiber distribution and compensate for rheological loss at low water-to-binder ratios, a dry-powder polycarboxylate ether (PCE) superplasticizer (BASF) was used.

### 2.2. Orthogonal Experimental Mix Design

To quantify the nonlinear influence of the w/b ratio on the toughness of multiscale fiber-reinforced concrete and to clarify the matrix-dependent synergy between SFs and CCWs, two reference matrices, designated Groups A and B, were developed according to close-packing principles and with reference to T/CBMF 37—2018 standard [36]. Table 3 presents the reference mix proportions.

The two matrices were deliberately differentiated by binder composition, aggregate proportion, and superplasticizer dosage to establish distinct matrix-density regimes. Specifically, Group A was designed to represent a highly compact matrix, incorporating quartz powder (7.50 kg/m^3^) and a higher fly ash content (180.00 kg/m^3^) to enhance particle packing, alongside a distinct fine-to-coarse aggregate ratio (771.75:415.60). This formulation enabled investigation within a low w/b ratio range. In contrast, Group B was formulated to provide a comparatively less compact matrix for evaluation within a higher w/b ratio range, achieved by omitting quartz powder (0.00 kg/m^3^), adjusting the cement/fly ash ratio (562.50: 112.50), and modifying its aggregate system (940.70: 386.50). This dual-matrix framework enables assessment of the reinforcing efficiency of CCWs and SFs across distinct matrix-density regimes, specifically allowing for the evaluation of the toughness-compensating capacity of higher fiber dosages in a less compact matrix.

For each matrix, a three-factor, four-level *L*_16_ (4^3^) orthogonal design was adopted. The investigated variables were w/b ratio, SF volume dosage (*V*_sf_), and CCW volume dosage (*V*_ccw_); their respective levels are listed in Table 4. This design permits an efficient evaluation of the individual effects of the three variables and their combined influence on toughness-related responses.

To capture the potential nonlinear effects of the w/b ratio, a targeted grouping strategy with a refined transition boundary was adopted. Group A covered a lower w/b ratio range of 0.22–0.31 in increments of 0.03, whereas Group B spanned 0.30–0.36 with a finer increment of 0.02. The narrow overlapping interval of 0.30–0.31 bridges the two groups, providing higher resolution across the transition from a dense to a more porous matrix. Furthermore, higher SF and CCW dosages were assigned to Group B to investigate whether enhanced crack-bridging and micro-arresting effects could compensate for the reduced matrix density at higher w/b ratios. The detailed orthogonal mix proportions are summarized in Table 5.

Groups A and B were intentionally differentiated not only by the w/b ratio, but also by binder composition and aggregate proportion. Therefore, the matrix-to-fiber transition inferred from the A–B comparison should be interpreted as the outcome of coupled effects across multiple design factors, rather than as evidence of a single-variable mechanism.

### 2.3. Specimen Preparation and Curing Protocol

A sequential mixing procedure was adopted to promote the uniform dispersion of SFs and CCWs. All mixtures were prepared using a forced-action planetary mixer. Fine and coarse aggregates were first dry-mixed for 30 s, after which cement, fly ash, silica fume, and quartz powder were added and mixed for a further 60 s. Subsequently, two-thirds of the mixing water, premixed with the full dosage of polycarboxylate superplasticizer, was added, and the mixture was mixed for 3 min. The CCWs and the remaining one-third of the water were then introduced, followed by an additional 3 min of mixing. Finally, the SFs were added gradually over 5 min to ensure uniform dispersion and prevent fiber balling.

The fresh mixtures were cast into oiled steel moulds and vibrated to remove entrapped air. Cubic specimens measuring 70.7 × 70.7 × 70.7 mm^3^ were prepared for compressive testing (Figure 2a), prismatic specimens measuring 40 × 40 × 160 mm^3^ for flexural testing (Figure 2b), and dog-bone-shaped specimens for direct tensile testing. The tensile test section had a cross-section of 15 × 30 mm^2^ (Figure 2c).

Immediately after casting, the exposed surfaces were covered with polyethylene film to prevent moisture loss. The specimens were demoulded after 24 h at 20 ± 2 °C and labeled. They were subsequently subjected to steam curing at 60–80 °C and 100% relative humidity for 24 h, followed by standard curing at 20 ± 2 °C and a relative humidity of at least 95% until testing at 28 days.

### 2.4. Mechanical Testing Program

The mechanical properties of the multiscale fiber-reinforced concrete were evaluated via quasi-static compressive, flexural, and direct uniaxial tensile tests, following GB/T 50081-2019 [37], GB/T 31387-2025 [38], and JC/T 2461-2018 [39]. Figure 2 schematically illustrates the specimen geometries, boundary conditions, and loading configurations.

For compressive strength, three 70 × 70 × 70 mm^3^ cubic specimens were tested for each mixture using an Instron 8805 electro-hydraulic servo-controlled testing system. Loading was applied under stress control at a constant rate of 0.6 MPa/s until macroscopic failure, and the maximum load was used to calculate the compressive strength (Figure 2a).

Flexural testing was conducted on three prismatic specimens (40 × 40 × 160 mm^3^) per mixture using an Instron 8802 servo-hydraulic testing system under three-point bending at a stress rate of 0.10 MPa/s over a support span of 100 mm (Figure 2b). Peak loads were recorded to compute the ultimate flexural strength and assess composite crack resistance under flexural tension.

Uniaxial direct tensile tests were performed on dog-bone-shaped specimens with a cross-section of 15 × 30 mm^2^ (Figure 2c) using the Instron 8802 system. Tests were conducted under displacement control at 0.5 mm/min to capture the complete post-cracking tensile response. To mitigate potential parasitic bending from secondary load eccentricity, two clip-on extensometers were mounted symmetrically on opposite faces of the gauge section, and their averaged output was used to determine tensile strain.

For all testing regimes, three valid replicate specimens were tested per mixture, and average values are reported alongside standard deviations.

## 3. Results and Analysis

### 3.1. Crack Evolution and Failure Characteristics

Figure 3 presents representative failure morphologies of SF–CCW reinforced concrete under compression, flexure, and direct tension. Across the three loading modes, the specimens exhibit a consistent damage sequence: cracks first develop in a distributed manner, then gradually localize into a dominant crack plane, and finally form a macrocrack while maintaining residual load transfer after the peak. This post-peak continuity is attributed to the crack-bridging effect of steel fibers and the crack-moderating influence of CCWs, which together prevent an abrupt separation-type failure.

The mix designs for the two experimental sets (A and B) differ mainly in cementitious composition and aggregate proportions (Table 3), and the test matrix spans different w/b ratio ranges (Table 4). Consequently, the extent and localization tendency of cracking vary with the w/b ratio, while the fiber-bridging mechanism remains the key factor sustaining the post-peak resistance.

#### 3.1.1. Compressive Failure

Under uniaxial compression, the cubic specimens (Figure 3a) exhibit a progressive failure characterized by initial defect compaction, followed by the development of distributed cracks that gradually localize. This leads to splitting-dominated crack patterns, often accompanied by inclined shear cracks. Post-peak, localized crushing and spalling occur at the edges and corners, but the specimens retain their overall form, indicating sustained load transfer. Fracture surfaces frequently show clear evidence of fiber bridging and pull-out, indicating their role in maintaining structural continuity.

#### 3.1.2. Flexural Failure

The SF–CCW-reinforced specimens subjected to flexure (Figure 3b) exhibit a quasi-ductile failure response. At the cracking load, microcracks initiate on the tension face and evolve into a dominant flexural crack that propagates toward the neutral axis. Beyond the peak load, this principal crack widens progressively, yet the specimens remain largely connected. This “cracked but unseparated” failure mode, characterized by gradual post-peak softening, is primarily sustained by the bridging action of steel fibers, undergoing debonding, slip, and pull-out across the crack plane.

#### 3.1.3. Direct Tensile Failure

Direct tensile tests on dumbbell-shaped specimens (Figure 3c) show failure localized within the gauge section, marked by the formation of a single dominant tensile crack. Initial microcracking, distributed through the matrix, eventually coalesces to form a through-crack at the weakest cross-section. Crucially, post-cracking resistance is maintained as stress transfers to steel fibers bridging the crack. The resulting fracture surfaces display matrix fracture alongside significant fiber debonding and pull-out, confirming that these mechanisms provide substantial energy dissipation and residual strength after crack localization.

Overall, all three loading conditions follow a common thread: the evolution from distributed micro-damage to localized macrocracks, followed by a sustained post-peak response. This behavior, departing significantly from brittle failure, highlights the critical role of multi-scale reinforcement. While calcium carbonate whiskers influence early-stage crack development, steel fibers primarily govern post-localization behavior by bridging macrocracks and ensuring continued load transfer. These characteristics depend on the specific mix proportions and constituent volume fractions, contributing to the nonlinear mechanical responses discussed in subsequent sections.

### 3.2. Analysis of the Evolution of Basic Mechanical Strength

This section comprehensively elucidates the 28-day compressive, flexural, and direct tensile strength performance of SF–CCW-reinforced concrete across varying w/b ratios, SF dosage, and CCW dosage (as detailed in mix design groups A and B). A central finding is that the strength responses under all loading modes exhibit significant non-monotonicity, rather than simple linear trends with respect to any single parameter. This underscores a complex interdependence among matrix quality, the synergistic actions of multi-scale reinforcing phases, and interfacial properties. These results further highlight the load-mode dependency in optimizing mix designs for different mechanical properties.

#### 3.2.1. Evolution of Compressive Strength

Figure 4 presents the raw measurements, mean values, and standard deviations of the 28-day compressive strength. The measured values ranged from approximately 82 to 140 MPa, with the maximum value recorded for A3 (140.44 MPa). Group A generally outperformed Group B; however, neither group exhibited a monotonic response to the water-to-binder ratio (w/b) or SFs-CCWs content. Instead, the results showed segmented fluctuations and local strength recovery. Group A increased from A1 to A3, then decreased and partially recovered at A7 and A12, whereas Group B generally declined from approximately 108 to 82 MPa, with a rebound at B11. The w/b ratio primarily determined matrix strength and the fiber–matrix interface, while the content and combination of steel fibers and calcium carbonate whiskers governed the local response. A relatively low w/b ratio promoted matrix densification and improved the conditions for whisker-based microcrack restraint and steel-fiber stress transfer. Calcium carbonate whiskers mainly refined local defects and impeded microcrack growth, whereas steel fibers restrained lateral deformation and delayed crack localization.

Nevertheless, excessive reinforcement or insufficient workability could cause whisker agglomeration, fiber clustering, entrapped air, or local paste deficiency, offsetting the benefits of a denser matrix. Thus, the compressive strength was governed by the coupled effects of matrix compactness, reinforcement dispersion, and interfacial stress transfer rather than by any single mixture parameter. The varying error-bar lengths further indicate that mixture design affected both strength and structural uniformity.

#### 3.2.2. Evolution of Flexural Strength

As shown in Figure 5, the flexural strengths of Groups A and B ranged from approximately 11.89–32.88 MPa and 13.50–23.51 MPa, respectively. Group A achieved the higher maximum strength but also showed greater scatter. A4 reached the highest value of 32.88 MPa, approximately 15.3% higher than A1, whereas the maximum compressive strength occurred at A3. This difference confirms that the optimum mixture for compression is not necessarily optimal for flexure. Unlike compression, flexural strength is particularly sensitive to tensile-zone defects and the efficiency of cross-crack stress transfer.

The strength increase from A1 to A4, followed by the sharp reduction at A5 and subsequent recovery at A7 and A8, demonstrates a clear non-monotonic response. Calcium carbonate whiskers can retard early crack growth through microscopic bridging, crack deflection, and crack-tip blunting. After macrocrack formation, steel fibers become the dominant load-transfer phase by bridging the crack. This sequential mechanism provides a continuous transition from microcrack control to macrocrack bridging.

However, excessive reinforcement or poor component compatibility may reduce workability and promote local defects, which act as stress concentrators and consequently reduce the observed flexural strength. Such defects are particularly detrimental because flexural failure is controlled by the weakest tensile section. The relatively high strength of B12 (23.51 MPa) suggests that, within a limited range, improved flowability at a higher w/b ratio may enhance SF-CCW dispersion and fiber bridging, leading to more effective stress transfer across potential crack planes and thus higher flexural strength. Nevertheless, this benefit cannot fully compensate for the associated increase in matrix porosity or loss of interfacial anchorage, both of which weaken the matrix and reduce the overall flexural capacity. The nonlinear flexural response therefore reflects the balance among matrix density, mixture rheology, reinforcement dispersion, and interfacial bonding, which collectively determine the material’s resistance to tensile failure under flexural loading.

#### 3.2.3. Evolution of Tensile Strength

Figure 6 shows that the 28-day direct tensile strengths ranged from 1.71 to 8.25 MPa. Group A exhibited higher overall strength than Group B, with values of approximately 3.48–8.25 MPa compared with 1.71–5.98 MPa. Considerable scatter was observed, reflecting the strong sensitivity of direct tensile strength to the weakest cross-section, local defects, and fiber orientation, all of which critically influence the material’s ability to resist direct tensile forces before macroscopic failure.

A4 recorded the highest strength, 8.25 MPa, approximately 29.5% higher than A1; A8 and A12 also reached relatively high values of 8.15 and 7.00 MPa, respectively. In contrast, A5 and A6 decreased to approximately 4 MPa. These results show that reducing the w/b ratio alone does not ensure higher tensile strength. Calcium carbonate whiskers primarily delay crack initiation by modifying microdefects, while steel fibers provide cross-crack bridging after crack localization. Their effectiveness depends on uniform dispersion and compatibility with the matrix’s rheology. Whisker agglomeration, fiber entanglement, or local paste deficiency can instead introduce defects and reduce bridging efficiency, leading to premature failure and lower measured tensile strength.

Group B exhibited similar local fluctuations, with relatively high values at B4, B14, and B16 and a minimum of 1.71 MPa at B5. The results indicate that improved reinforcement dispersion may partially compensate for the adverse effects of higher porosity and weaker interfaces, allowing the material to achieve better tensile strength even under less ideal matrix conditions. However, this compensation is conditional and does not increase monotonically with reinforcement content, suggesting that an optimal balance is required to maximize direct tensile strength.

Overall, the w/b ratio controlled the basic load-bearing capacity of the matrix and interface, whereas calcium carbonate whiskers and steel fibers acted primarily at the stages of crack initiation and macrocrack propagation, respectively. The different strength maxima obtained under compression, flexure, and tension demonstrate a load-mode-dependent component-matching effect. Effective mixture design should therefore balance matrix compactness, reinforcement dispersion, workability, and interfacial anchorage rather than simply minimizing the w/b ratio or maximizing reinforcement content.

### 3.3. Effects of Constituent Factors on Basic Mechanical Properties

#### 3.3.1. Compressive Strength

Figure 7 and Figure 8 identify water content as the principal factor governing compressive strength. As water content increased from 165 to 232.5 kg/m^3^, the mean strength of Group A decreased from 133.82 to 96.66 MPa (27.8%). Group B showed a smaller reduction of approximately 16%, from 106.69 to 89 MPa, over 225–270 kg/m^3^. The decline is primarily attributable to increased capillary porosity and deterioration of the paste and interfacial transition zones. The greater sensitivity of Group A indicates that pore-structure changes exert a stronger influence within the lower-water-content range.

SFs had a comparatively modest, non-monotonic effect. Group A reached approximately 120.41 MPa at 157.0 kg/m^3^ and subsequently remained nearly stable, whereas Group B peaked at 100.64 MPa at 196.25 kg/m^3^ before declining. Moderate fiber incorporation restrained lateral deformation and delayed crack localization; however, excessive dosages could promote fiber clustering, air entrapment, and local paste deficiency, offsetting these benefits.

CCWs exhibited a clearer dosage threshold. The maximum strengths occurred at 37.66 kg/m^3^ in Group A (120.62 MPa) and 40.35 kg/m^3^ in Group B (99.81 MPa), followed by decreases at higher dosages. Moderate CCW dosage improved matrix continuity through microfilling, crack-tip pinning, and microscale bridging, whereas excessive CCW dosage increased water demand and the risk of agglomeration. Thus, water content controlled the overall compressive strength, while SFs and CCWs introduced nonlinear effects through crack restraint and interfacial regulation.

#### 3.3.2. Flexural Strength

Figure 9 and Figure 10 show that flexural strength was more sensitive to constituent variation than compressive strength. In Group A, increasing water content from 165 to 232.5 kg/m^3^ reduced the mean flexural strength from 28.43 to 14.38 MPa, corresponding to a 49.4% loss. This pronounced reduction reflects the tensile zone’s sensitivity to pores and interfacial defects. By contrast, Group B varied only from 17.48 to 18.88 MPa, suggesting a competition between matrix weakening and improved reinforcement dispersion at higher water contents.

SFs provided the most consistent improvement. Increasing their dosage from the lowest to the highest level raised the flexural strength from 18.02 to 24.42 MPa in Group A and from 14.75 to 21.28 MPa in Group B, representing gains of 35.5% and 44.3%, respectively. This enhancement resulted from crack bridging and energy dissipation through interfacial debonding, frictional sliding, and pull-out.

The CCW effect was threshold-dependent. Group A reached 22.38 MPa at 37.66 kg/m^3^ and then stabilized, whereas Group B peaked at 19.83 MPa at 40.35 kg/m^3^ before declining. Moderate CCW dosages delayed microcrack initiation and complemented steel-fiber bridging, establishing a multiscale pathway from microcrack arrest to macrocrack control. Excessive CCWs impaired rheology and dispersion, weakening this synergy. Flexural performance was therefore governed by the balance among matrix compactness, CCW dispersion, and steel-fiber bridging.

#### 3.3.3. Direct Tensile Strength

Figure 11 and Figure 12 demonstrate the high compositional sensitivity of direct tensile strength. In Group A, increasing water content from 165 to 232.5 kg/m^3^ reduced the mean strength from 7.31 to 4.13 MPa (43.5%), owing to increased porosity and weakened interfacial bonding. Group B decreased from 4.59 to 3.43 MPa before recovering to 4.82 MPa. This rebound likely reflects improved workability and reinforcement dispersion rather than a direct benefit of higher water content.

SFs increased tensile strength from 4.92 to 6.84 MPa in Group A and from 3.55 to 4.83 MPa in Group B. The greater improvement at higher dosages suggests that effective stress redistribution requires a sufficiently dense crack-bridging network. The observed scatter nevertheless confirms the strong dependence of direct tensile strength on fiber orientation, spatial distribution, and the weakest cross-section.

CCWs primarily affected crack initiation. Group A increased from 4.72 to approximately 5.7 MPa before reaching a plateau, whereas Group B initially decreased to 3.28 MPa and subsequently recovered to approximately 4.65 MPa. Their effectiveness was therefore constrained by matrix rheology and dispersion quality. CCWs can delay microcrack initiation but cannot replace steel fibers in transferring stress across localized macrocracks.

Overall, the w/b ratio governed matrix and interfacial quality, CCWs controlled early microcracking, and SFs dominated macrocrack bridging. Their differing effects across loading modes demonstrate that multiscale reinforcement arises from load-dependent constituent matching rather than simple additive strengthening. Because the plotted means also incorporate other factor levels, analysis of variance and interaction-effect testing would verify the significance of local variations.

The current study used a single type of steel fiber with a specific geometry (l/d = 64, l = 12.8 mm). While the findings show the dominant role of SF bridging in the higher w/b range for this specific fiber, the effectiveness of steel fibers in flexural and tensile behavior depends heavily on parameters such as fiber shape, aspect ratio, and volume fraction [40]. Future research could explore how these different fiber characteristics influence the observed mechanism transition and overall performance in similar concrete systems.

### 3.4. Range Analysis: W/B Ratio Level-Dependent Contributions of Matrix and Multiscale Fibers

The range analysis in Table 6 compares the relative influence of the w/b ratio, SFs, and CCWs on the orthogonally measured strength responses of Groups A and B. Overall, the dominant factor varies across loading modes, and the governing contribution of each component depends on the w/b ratio level.

For compressive strength, water content exhibits the largest ranges in both groups (R = 37.16 for Group A and R = 16.67 for Group B), clearly exceeding the ranges of SFs and CCWs. This indicates that compression is primarily governed by the matrix condition and the quality of the interfacial transition, whereas multiscale reinforcements act primarily as secondary modifiers. For flexural strength, the controlling factor changes with the w/b level. In Group A, water content remains dominant (R = 14.05), whereas in Group B, the SFs become the most influential factor (R = 6.45), in contrast to the much smaller effect of water (R = 1.40). A similar tendency appears in direct tensile strength, where SFs show the largest range in Group B (R = 1.40). Together, these results point to a load-transfer transition: under different matrix conditions, the balance shifts from matrix-controlled strength development toward fiber-governed bridging and post-cracking resistance.

The level-mean trends further clarify the roles of the two reinforcing phases. Increasing SF content improves flexural and tensile strengths, with the most pronounced response in Group B; for flexure, the mean increases from k_1_ c 14.75 MPa to k_4_ = 21.21 MPa. By contrast, CCWs generally show an intermediate optimum. In Group A, the compressive and flexural means peak at k_3_ (120.62 MPa and 22.31 MPa, respectively), then decrease at k_4_, suggesting that excessive CCWs may reduce overall effectiveness by lowering reinforcement dispersion efficiency and increasing sensitivity to local defects. Water content also exhibits non-monotonic behavior in the tensile response in Group B (the mean varies from 4.59 to 3.86, then to 3.63, and finally rebounds to 4.82 MPa as the water level increases). This pattern implies competing effects of water on matrix condition and fresh-mixture workability. However, because an orthogonal design cannot directly isolate fiber dispersion and interfacial interactions, the observed nonlinearity should be regarded as an empirical indication of regime-dependent response rather than direct proof of a specific interaction mechanism.

Based on the range and mean trends, a practical candidate combination for achieving a balanced improvement is k_water_ = 1, k_SF_ = 4, and k_CCW_ = 3. Confirm this selection with dedicated verification tests before treating it as the overall optimum.

### 3.5. Analysis of Variance: Factorial Dominance and Mechanistic Transitions

The Analysis of Variance (ANOVA) results in Table 7 are consistent with the trends obtained from the range analysis, demonstrating that the governing contribution of mixture factors depends on both the w/b ratio regime (Group A vs. Group B) and the loading mode.

For compressive strength, the w/b ratio exhibits a clear and statistically significant effect in Group A (F = 31.37, *p* = 0.0005), while the main effects of SFs and CCWs are not significant (*p* = 0.144 and *p* = 0.186, respectively). In Group B, none of the three factors reach statistical significance; notably, the influence of the w/b ratio weakens (*p* = 0.0679), indicating that compressive performance is not uniformly controlled by matrix-related parameters across regimes. This shift supports a dosage-dependent transition in how load transfer occurs under compression.

For flexural strength, the controlling factor changes more distinctly between the two groups. In Group A, the w/b ratio is significant (F = 19.89, *p* = 0.0016), whereas SFs and CCWs show no significant main effects (*p* = 0.0566 and *p* = 0.2502). In Group B, SFs become the dominant statistically significant factor (F = 16.34, *p* = 0.0027), while the w/b ratio and CCWs do not show significant effects (*p* = 0.5838 and *p* = 0.1534). This regime-dependent reversal provides direct statistical support for the load-mode-dependent matching between matrix control and fiber bridging.

For tensile strength, the ANOVA indicates that none of the tested main effects are statistically significant in either group (all *p*-values are above the conventional threshold). This does not imply that reinforcement is ineffective; rather, it suggests that tensile response is governed by a more distributed set of microstructural variables (e.g., scatter in local reinforcement distribution and interfacial conditions) that a main-effect-only model cannot resolve at the present experimental resolution.

Overall, Table 7 presents a clear statistical picture: under compression, matrix-related w/b ratio effects dominate only in Group A; under flexure, water controls in Group A, whereas SFs dominate in Group B. For tension, tensile strength appears to be controlled by coupled factors not captured by single-factor main effects. Together with the range analysis, these results support a regime-dependent governing mechanism rather than a universal single-parameter design rule.

## 4. Mechanistic Interpretation and Mix-Design Implications

### 4.1. Hierarchical Toughening Through Cross-Scale Reinforcement

Combined insights from range analysis and ANOVA clearly show that SFs and CCWs contribute sequentially and complementarily to concrete fracture resistance. This forms a hierarchical toughening mechanism characterized by whisker-induced microcrack control, followed by steel-fiber bridging, both intricately governed by matrix-dependent interfacial interactions.

(1) Whisker-Induced Microcrack Control: The Critical Dosage Threshold

CCWs primarily act at the microscale, mitigating local defects, bridging nascent microcracks, and blunting crack tips. Together, these actions enhance the cementitious matrix’s inherent resistance to crack initiation and delay crack coalescence. Crucially, however, their contribution is exquisitely dosage-sensitive. Given their high specific surface area, excessive whisker content invariably promotes agglomeration, which in turn compromises local particle packing and can introduce detrimental weak interfaces or stress concentrations. This interpretation aligns perfectly with the non-monotonic response observed in the range analysis; for instance, the mean compressive strength in Group A peaked at k_3_ (120.62 MPa) and then declined at k_4_ (112.56 MPa). The statistically insignificant main effects for whiskers identified in the ANOVA (all *p* > 0.15) do not, therefore, denote ineffectiveness. Rather, they underscore that the whisker’s performance cannot be adequately captured by a simple monotonic relationship over the investigated dosage range, strongly implying a critical optimum. This observed threshold behavior, in which beneficial effects are offset by deleterious ones beyond an ideal concentration, highlights the need for statistical modeling, such as quadratic or interaction-based analyses, to fully characterize whisker efficacy.

(2) SFs Bridging: Sustaining Post-Cracking Integrity

Millimeter-scale SFs play a key role in governing crack propagation once matrix cracking occurs. SFs that bridge a crack effectively transfer tensile stresses and dissipate fracture energy through a multifaceted combination of interfacial debonding, frictional pull-out, and mechanical anchorage. Consequently, we observed a clear trend of increasing mean flexural and tensile strengths with higher SF dosages. In Group B, for instance, the flexural strength robustly increased from 14.75 MPa at k_1_ to 21.21 MPa at k_4_. The statistical significance of SFs on the flexural strength of Group B (F = 16.34, *p* = 0.0027) unequivocally confirms their dominant role under these matrix conditions. This result signifies a fundamental transition: from matrix-controlled cracking to a fiber-controlled post-cracking resistance. An optimally developed SF network is thus indispensable for enhancing load transfer across macro-cracks and for delaying unstable fracture, particularly under flexural and direct tensile loading.

(3) Matrix-Dependent Cross-Scale Interaction: A Balancing Act

The two distinct reinforcement scales establish a sophisticated hierarchical crack-control system: CCWs govern microcrack initiation and early-stage propagation, whereas SFs effectively bridge localized macrocracks and sustain load transfer as cracks open progressively. However, the efficacy of this combined action depends strongly on the underlying matrix characteristics. The w/b ratio, for example, not only dictates capillary porosity and matrix strength but also profoundly influences the fresh-state rheology, the uniformity of fiber dispersion, and the quality of the ITZ. An excessively low w/b ratio, while promoting matrix densification, can simultaneously impede uniform fiber dispersion due to high mixture viscosity. Conversely, an elevated ratio improves workability but risks increased porosity and compromised fiber–matrix bonding. The ultimate mechanical response thus depends on a delicate, nonlinear balance among matrix compactness, reinforcement dispersion, and interfacial stress transfer, rather than the simple, independent contribution of any single constituent.

### 4.2. Matrix-Dependent Optimization: Engineering Implications for High-Performance Concrete

The statistical analyses unequivocally demonstrate that the governing strengthening mechanisms evolve with changes in matrix and fiber conditions. This finding fundamentally challenges conventional “more-is-better” approaches to mix design, advocating instead for a sophisticated, coordinated control strategy that judiciously balances matrix quality, SF bridging capacity, and CCW dosage.

(1) Matrix-Dominated Regime: Prioritizing Compactness and Microscale Control

In compositions corresponding to Group A, where the w/b ratio significantly affected both compressive (F = 31.37, *p* = 0.0005) and flexural strengths (F = 19.89, *p* = 0.0016), matrix compactness emerges as the primary design objective. In this regime, maintaining a low w/b ratio is critical, provided that sufficient workability is preserved to ensure homogeneous dispersion of the reinforcement. Our results suggest an intermediate CCW dosage (near k_3_) and a moderate-to-high SF dosage (k_3_–k_4_) for optimal performance. Crucially, high paste viscosity increases the risk of clustering or entrapped defects, so excessive reinforcement levels should be avoided.

(2) Fiber-Dominated Regime: Maximizing Post-Cracking Resistance

Conversely, in compositions analogous to Group B, where the w/b ratio’s influence on flexural strength became statistically insignificant (*p* = 0.5838) but SFs exerted a highly significant effect, the design priority shifts dramatically. Here, the paramount objective is to establish an effective SF bridging network, with the highest investigated dosage (k_4_) delivering superior flexural resistance. In this context, CCWs can still serve as a valuable complement by mitigating microscale defects, but their dosage should be kept near the identified k3 optimum to avoid agglomeration-induced deleterious effects.

(3) Engineering Practicalities and Holistic Design

These mechanistic insights call for a refined engineering approach to multi-scale reinforced concrete. Design should follow a matrix–fiber compatibility principle: the w/b ratio governs matrix integrity, SFs dictate post-cracking toughness, and CCWs precisely regulate microscale damage within a judiciously constrained dosage window. For structural elements primarily subjected to compression, focus on maximizing matrix densification and controlling defects. However, for applications where flexure, tension, impact, or cyclic loading are dominant–such as pavements, beams, or specialized structures–a robustly connected SF network, combined with an optimal CCW dosage and appropriate rheological control, becomes indispensable. This hierarchical, performance-based design strategy offers a rational and scientifically sound pathway to achieving an optimal balance among strength, toughness, and practical constructability.

## 5. Conclusions

Using two orthogonal experimental groups spanning distinct w/b-ratio ranges, this study clarifies how the dominant reinforcement mechanism in SF–CCW concrete evolves with the matrix state. The main conclusions are as follows.

(1) Matrix-state-dependent mechanism transition. The two groups represent successive stages of a single evolving toughening process. At low w/b ratio (e.g., 0.22–0.31), matrix compactness governs strength. As the w/b ratio increases (e.g., above 0.34) and matrix resistance weakens, load transfer shifts toward fiber bridging, with SFs controlling flexural capacity.

(2) Cooperative cross-scale crack control. CCWs primarily reduce microcrack initiation by micro-filling and crack-tip blunting, while SFs sustain post-cracking resistance by bridging and interfacial pull-out/friction. Together, the failure mode evolves from brittle fracture to progressive ductile cracking.

(3) Non-identical dosage–performance relationships. SF contribution increases monotonically with dosage, whereas CCW efficiency peaks and declines beyond a threshold due to agglomeration-related deterioration. This indicates that macro- and micro-scale reinforcements require different design logic for maximum effectiveness.

(4) The w/b ratio acts as an indirect regulator of reinforcement efficacy. Beyond strengthening the matrix, the w/b ratio governs porosity, interfacial bonding, and fiber dispersion, thereby modulating the effectiveness of both SFs and CCWs. This coupling explains why identical fiber dosages yield different outcomes under varying matrix conditions.

(5) Implication for zone-specific mix design. For compact, low-w/b-ratio (e.g., below 0.31) matrices, the priority is to maintain matrix density while ensuring adequate fiber dispersion. For more porous, crack-prone matrices (e.g., w/b ratio above 0.34), SFs should be increased to enhance post-cracking bridging, while CCW content should be limited within its effective dosage window (e.g., below 2.0% by volume). The key design principle is coordinated balance among matrix integrity, microscale crack resistance, and macroscale toughening, rather than maximizing a single component.

(6) The identified dominant reinforcement mechanisms, particularly the governing role of SF bridging at higher w/b ratios, are specifically demonstrated for the investigated steel fiber geometry (l/d = 64, l = 12.8 mm). Further studies are needed to explore how fiber type and geometry affect these mechanisms.

## Figures and Tables

**Figure 1:**
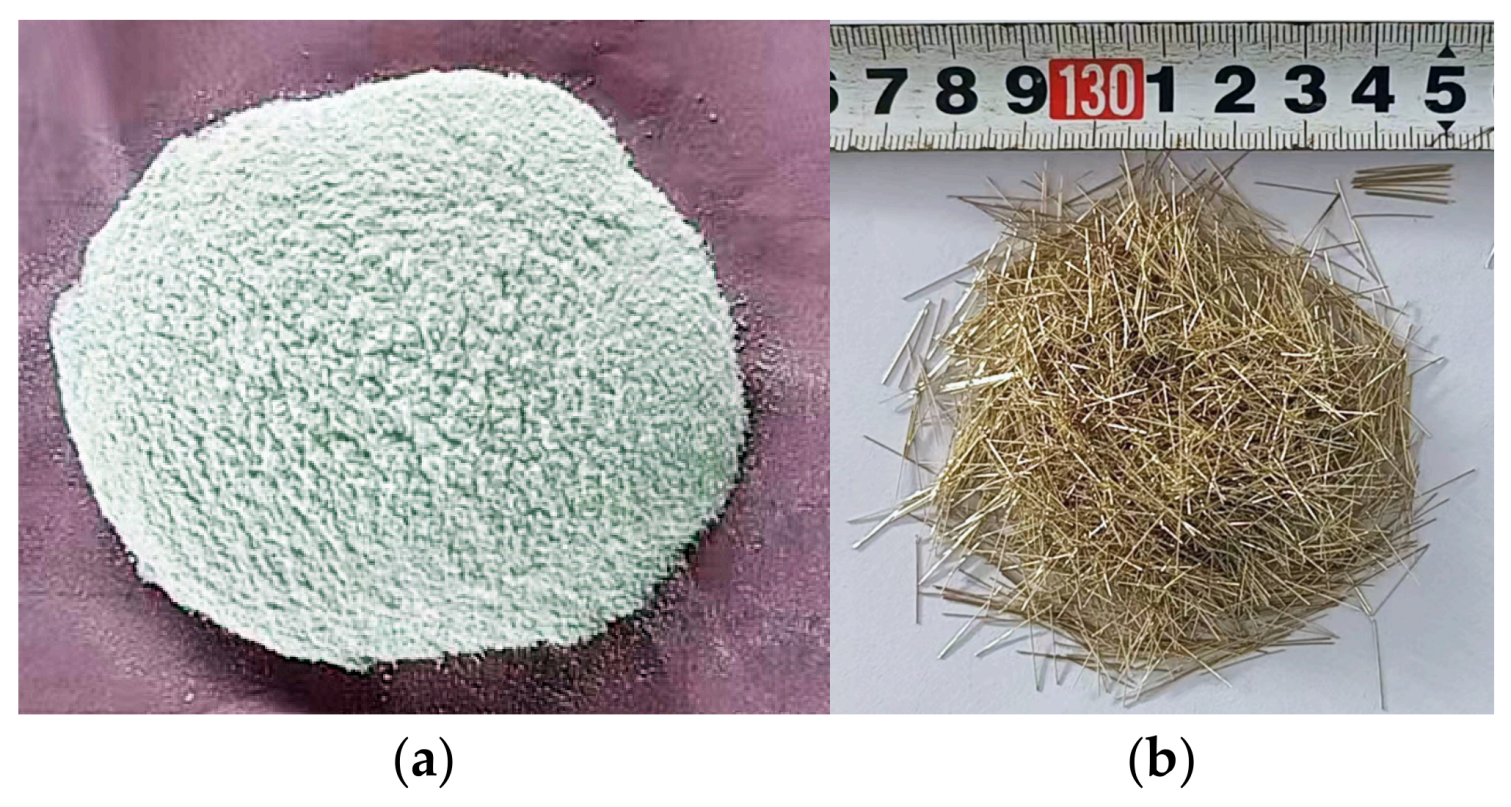
Photographs of reinforcing materials: (**a**) CaCO_3_ whiskers; (**b**) steel fiber.

**Figure 2:**
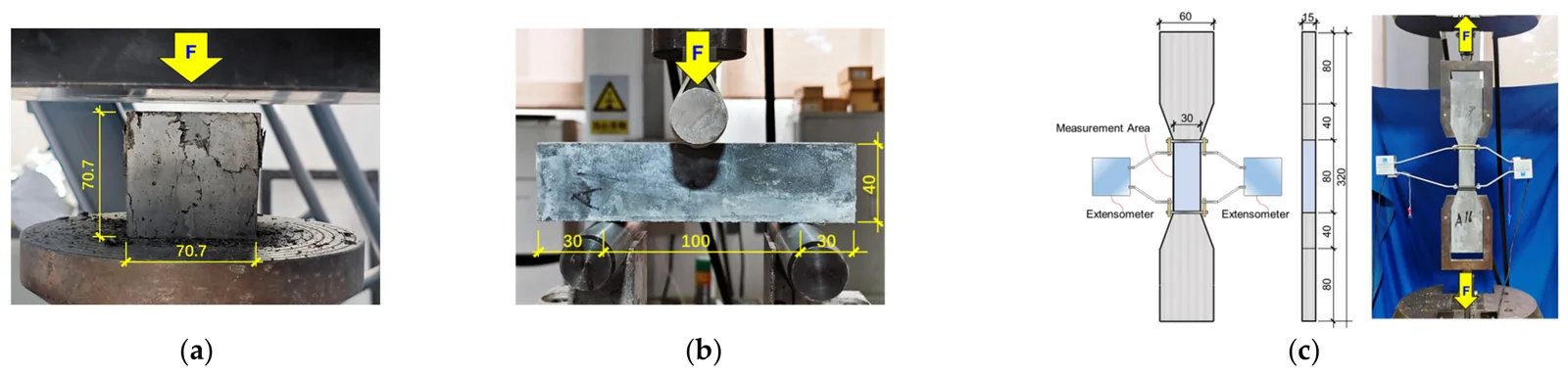
Specimen geometries and experimental setups for mechanical testing: (**a**) compression; (**b**) flexure; and (**c**) direct tension.

**Figure 3:**
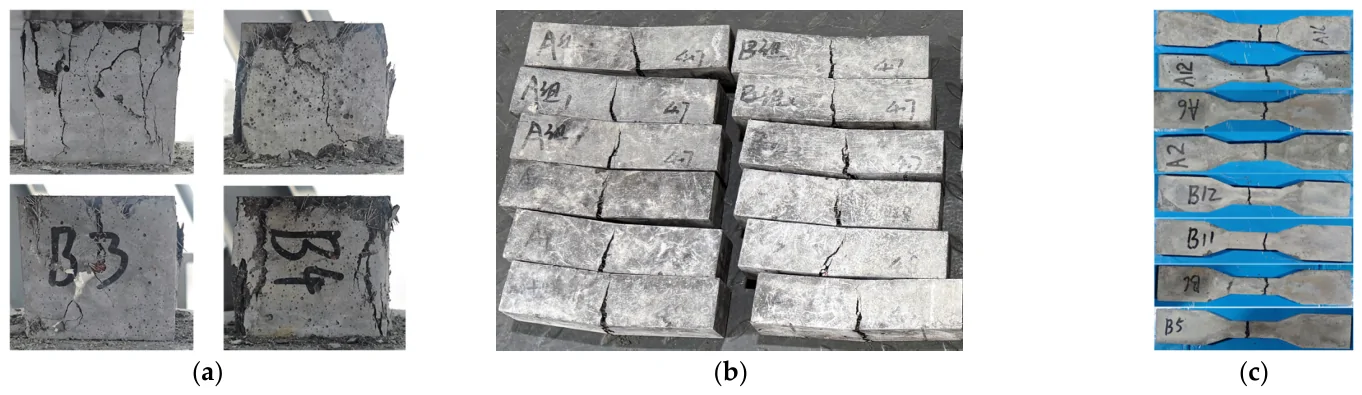
Typical failure modes of specimens under different mechanical tests: (**a**) compressive failure; (**b**) flexural failure; (**c**) direct tensile failure.

**Figure 4:**
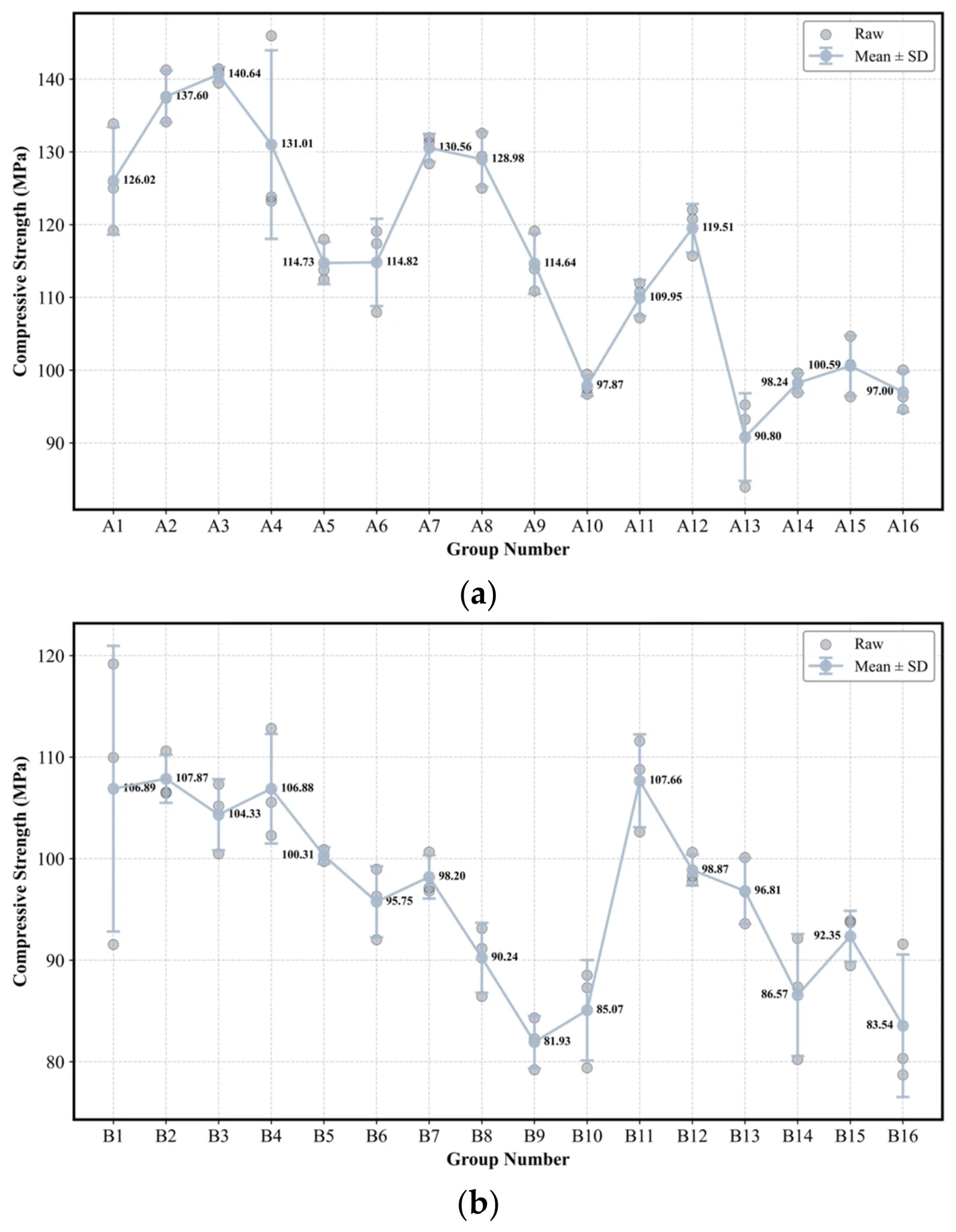
Compressive strength: (**a**) Group A; (**b**) Group B.

**Figure 5:**
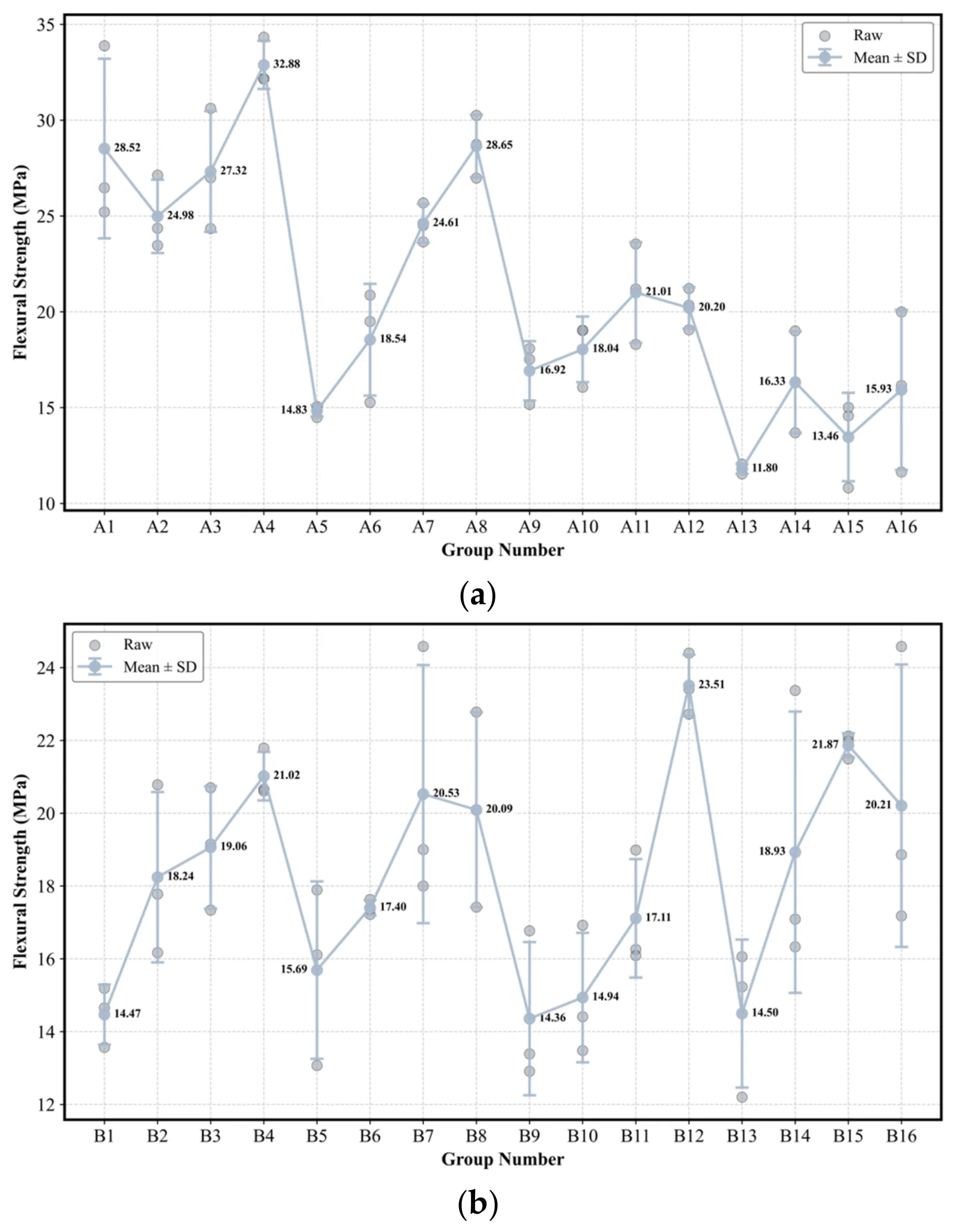
Flexural strength: (**a**) Group A; (**b**) Group B.

**Figure 6:**
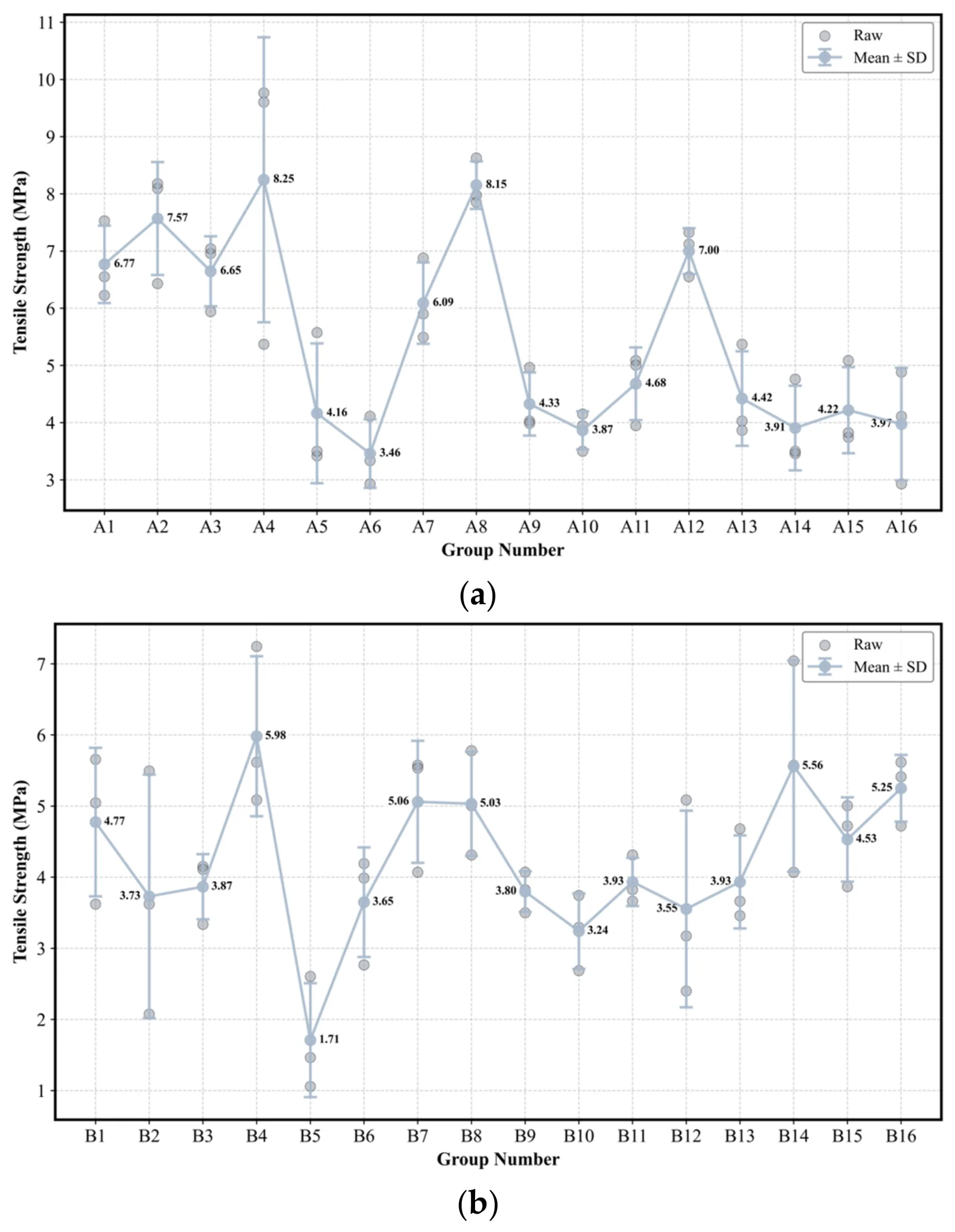
Tensile strength: (**a**) Group A; (**b**) Group B.

**Figure 7:**
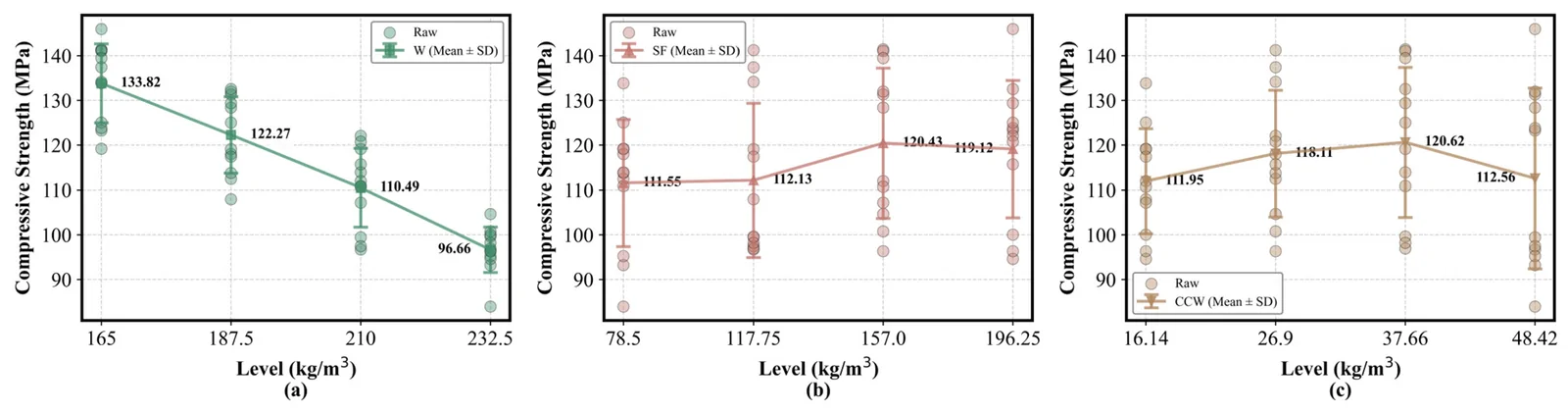
Variation in compressive strength with main factors in Group A: (**a**) water; (**b**) steel fiber; (**c**) CCW.

**Figure 8:**
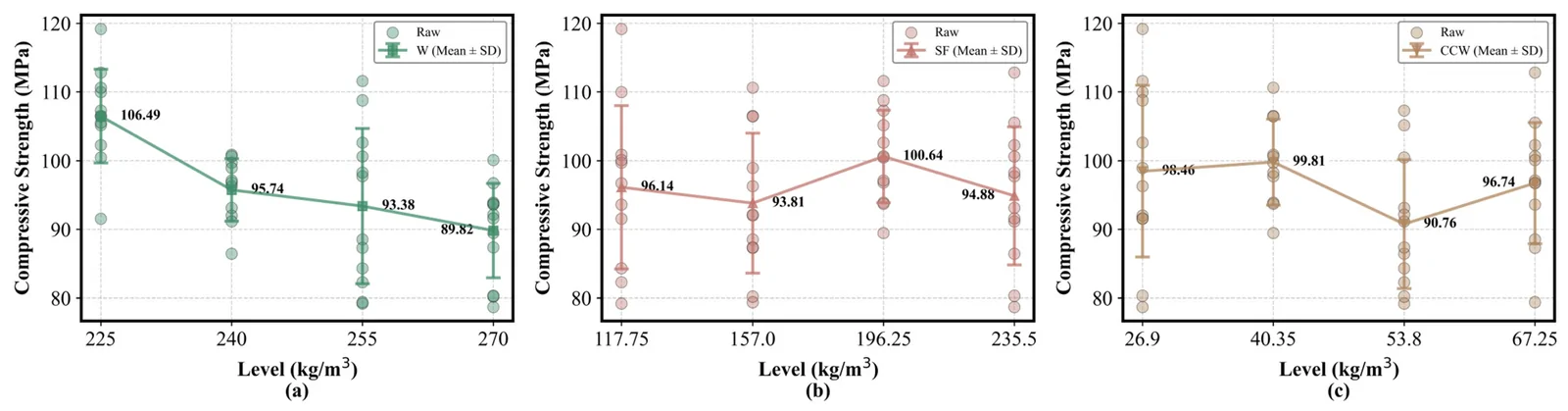
Variation in compressive strength with main factors in Group B: (**a**) water; (**b**) steel fiber; (**c**) CCW.

**Figure 9:**
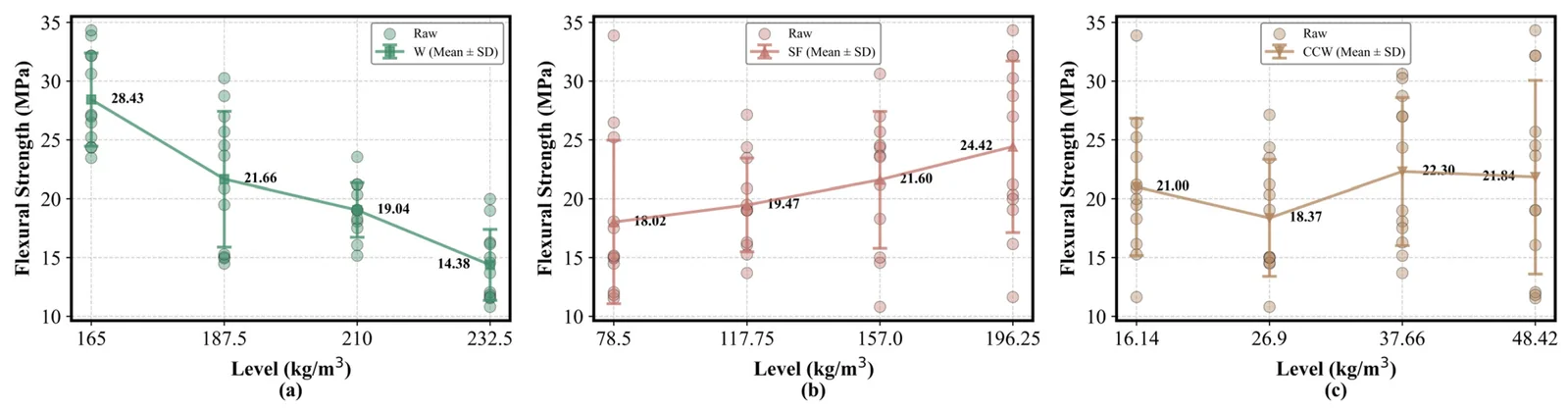
Variation in flexural strength with main factors in Group A: (**a**) water; (**b**) steel fiber; (**c**) CCW.

**Figure 10:**
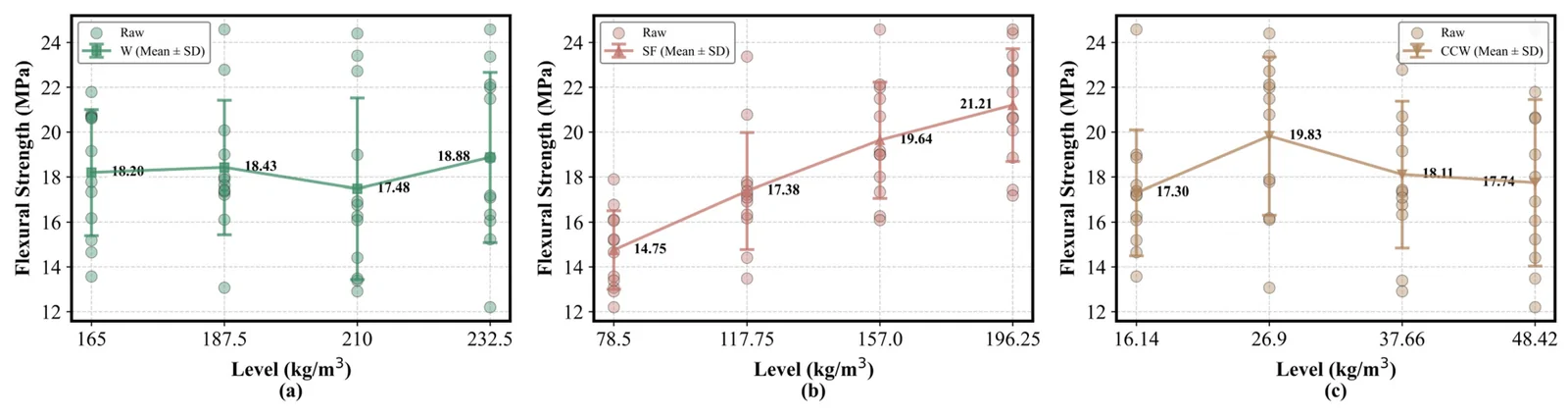
Variation in flexural strength with main factors in Group B: (**a**) water; (**b**) steel fiber; (**c**) CCW.

**Figure 11:**
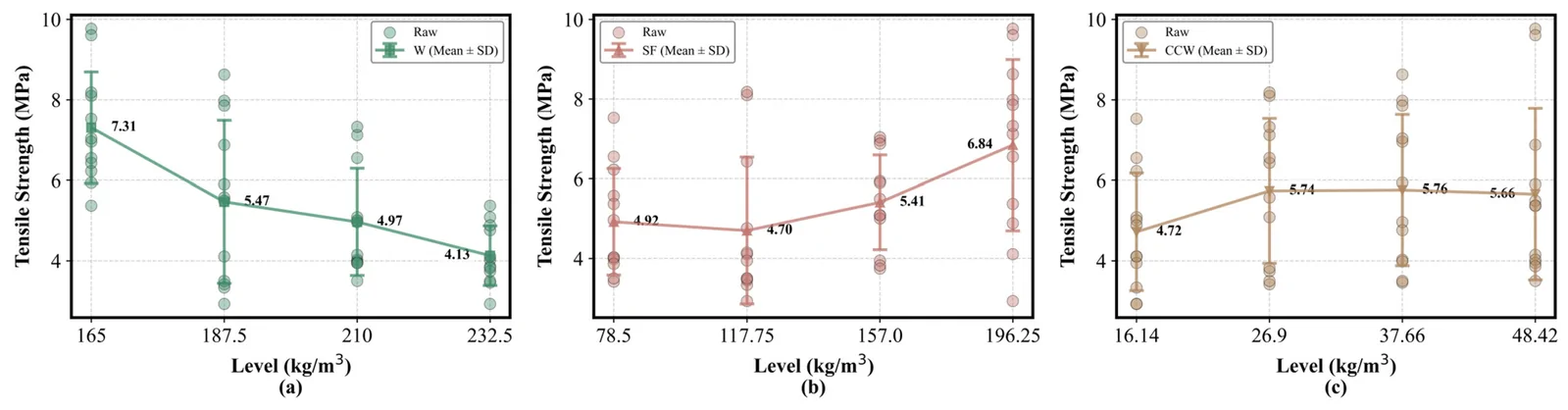
Variation in tensile strength with main factors in Group A: (**a**) water; (**b**) steel fiber; (**c**) CCW.

**Figure 12:**
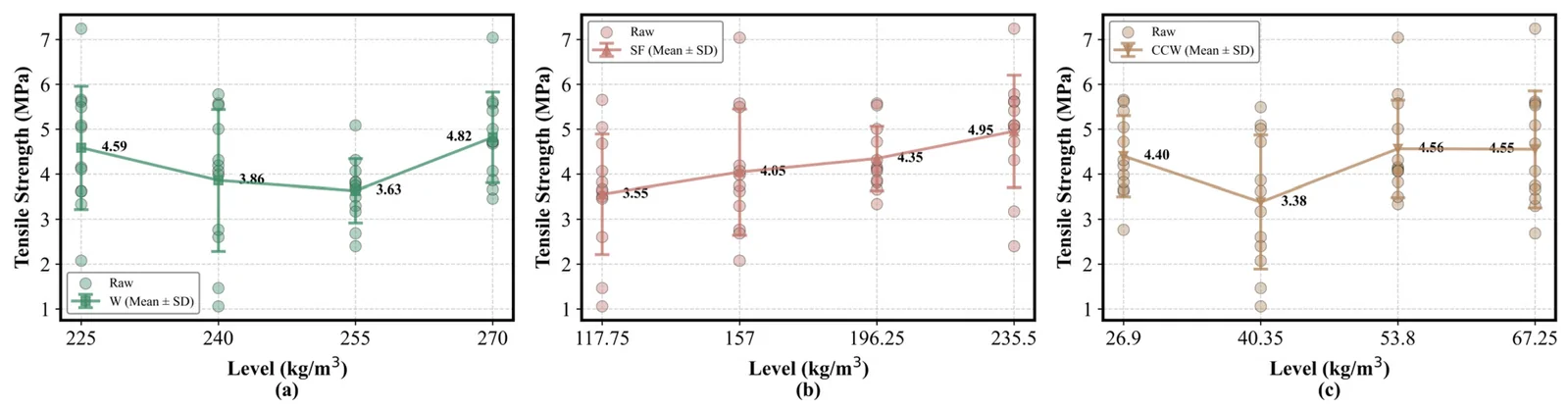
Variation in tensile strength with main factors in Group B: (**a**) water; (**b**) steel fiber; (**c**) CCW.

**Table 1:** Physical property parameters of calcium carbonate whiskers [35].

Purity (%)	Moisture Content (%)	Equivalent Diameter (µm)	Length-to-Diameter Ratio	pH	Heat Resistance (°C)
98	0.38	8	21	6.5	980

**Table 2:** Physical property parameters of steel fiber.

Tensile Strength (MPa)	Length (mm)	Equivalent Diameter (mm)	Length-to-Diameter Ratio
2988	12.8	0.2	64

**Table 3:** Reference mix proportions (kg/m^3^).

Group	Cement	Fly Ash	Silica Fume	Quartz Powder	Fine Aggregate (Sand)	Coarse Aggregate	Superplasticizer
A	487.50	180.00	75.00	7.50	771.75	415.60	13.05
B	562.50	112.50	75.00	0.00	940.70	386.50	7.25

**Table 4:** Orthogonal experimental factors and levels for Experimental Groups A and B.

Test Group	Level	Factor 1: w/b Ratio	Factor 2: SF *V*_sf_ (%)	Factor 3: CCW *V*_ccw_ (%)
A	1	0.22	1.0	0.6
	2	0.25	1.5	1.0
	3	0.28	2.0	1.4
	4	0.31	2.5	1.8
B	1	0.30	1.5	1.0
	2	0.32	2.0	1.5
	3	0.34	2.5	2.0
	4	0.36	3.0	2.5

**Table 5:** Experimental Groups A and B: orthogonal test mix proportions (kg/m^3^).

Test Group	No.	Water	SF	CCW
A	A1	165.00	78.50 (1.0)	16.14 (0.6)
	A2	165.00	117.75 (1.5)	26.90 (1.0)
	A3	165.00	157.00 (2.0)	37.66 (1.4)
	A4	165.00	196.25 (2.5)	48.42 (1.8)
	A5	187.50	78.50 (1.0)	26.90 (1.0)
	A6	187.50	117.75 (1.5)	16.14 (0.6)
	A7	187.50	157.00 (2.0)	48.42 (1.8)
	A8	187.50	196.25 (2.5)	37.66 (1.4)
	A9	210.00	78.50 (1.0)	37.66 (1.4)
	A10	210.00	117.75 (1.5)	48.42 (1.8)
	A11	210.00	157.00 (2.0)	16.14 (0.6)
	A12	210.00	196.25 (2.5)	26.90 (1.0)
	A13	232.50	78.50 (1.0)	48.42 (1.8)
	A14	232.50	117.75 (1.5)	37.66 (1.4)
	A15	232.50	157.00 (2.0)	26.90 (1.0)
	A16	232.50	196.25 (2.5)	16.14 (0.6)
B	B1	225.00	117.75 (1.5)	26.90 (1.0)
	B2	225.00	157.00 (2.0)	40.35 (1.5)
	B3	225.00	196.25 (2.5)	53.80 (2.0)
	B4	225.00	235.50 (3.0)	67.25 (2.5)
	B5	240.00	117.75 (1.5)	40.35 (1.5)
	B6	240.00	157.00 (2.0)	26.90 (1.0)
	B7	240.00	196.25 (2.5)	67.25 (2.5)
	B8	240.00	235.50 (3.0)	53.80 (2.0)
	B9	255.00	117.75 (1.5)	53.80 (2.0)
	B10	255.00	157.00 (2.0)	67.25 (2.5)
	B11	255.00	196.25 (2.5)	26.90 (1.0)
	B12	255.00	235.50 (3.0)	40.35 (1.5)
	B13	270.00	117.75 (1.5)	67.25 (2.5)
	B14	270.00	157.00 (2.0)	53.80 (2.0)
	B15	270.00	196.25 (2.5)	40.35 (1.5)

Note: Values in parentheses indicate the corresponding volume fractions (vol%) of SFs and CCWs.

**Table 6:** Range analysis of factor effects on compressive, flexural, and tensile strength.

Mechanical Performance	Test Group	Level	Water	SF	CCW
Compressive strength	Group A	k_1_	133.82	111.55	111.95
		k_2_	122.27	112.13	118.11
		k_3_	110.49	120.43	120.62
		k_4_	96.66	119.12	112.56
		R	37.16	8.88	8.68
	Group B	k_1_	106.49	96.48	98.46
		k_2_	96.12	93.81	99.85
		k_3_	93.38	100.64	90.76
		k_4_	89.82	94.88	96.74
		R	16.67	6.82	9.09
Flexural strength	Group A	k_1_	28.43	18.02	21.00
		k_2_	21.66	19.47	18.37
		k_3_	19.04	21.60	22.31
		k_4_	14.38	24.42	21.84
		R	14.05	6.40	3.93
	Group B	k_1_	18.20	14.75	17.30
		k_2_	18.43	17.38	19.83
		k_3_	17.48	19.64	18.11
		k_4_	18.87	21.21	17.74
		R	1.40	6.45	2.53
Tensile strength	Group A	k_1_	7.31	4.92	4.72
		k_2_	5.47	4.70	5.74
		k_3_	4.97	5.41	5.76
		k_4_	4.13	6.84	5.66
		R	3.18	2.14	1.04
	Group B	k_1_	4.59	3.55	4.40
		k_2_	3.86	4.05	3.38
		k_3_	3.63	4.35	4.56
		k_4_	4.82	4.95	4.55
		R	1.19	1.40	1.18

Note: k_1_ to k_4_ represent the average performance at each factor’s level 1 to 4, respectively; R denotes the range, indicating the influence of the factor.

**Table 7:** Analysis of Variance (ANOVA) of Mixture Factors on Mechanical Performance.

Index	Group	Factor	SS	DOF	MS	F	*p*	Significance
Compressive strength	A	Water	3043.97	3	1014.66	31.37	0.0005	***
		SF	256.05	3	85.35	2.64	0.144	-
		CCW	215.88	3	71.96	2.22	0.186	-
		Error	194.09	6	32.35	-	-	-
	B	Water	617.35	3	205.78	4.07	0.0679	-
		SF	107.67	3	35.89	0.71	0.5807	-
		CCW	192.07	3	64.02	1.27	0.3673	-
		Error	303.45	6	50.58	-	-	-
Flexural strength	A	Water	412.74	3	137.58	19.89	0.0016	**
		SF	92.77	3	30.92	4.47	0.0566	-
		CCW	37.01	3	12.34	1.78	0.2502	-
		Error	41.51	6	6.92	-	-	-
	B	Water	4.08	3	1.36	0.7	0.5838	-
		SF	94.69	3	31.56	16.34	0.0027	**
		CCW	14.67	3	4.89	2.53	0.1534	-
		Error	11.59	6	1.93	-	-	-
Tensile strength	A	Water	21.70	3	7.23	6.32	0.0622	-
		SF	11.14	3	3.71	3.24	0.1261	-
		CCW	3.01	3	1.00	0.88	0.2835	
		Error	6.87	6	1.14	-	-	-
	B	Water	3.87	3	1.29	1.68	0.1825	-
		SF	4.12	3	1.37	1.79	0.7331	-
		CCW	3.87	3	1.29	1.68	0.5612	-
		Error	4.61	6	0.77	-	-	-

Note: ** indicates statistical significance at *p* < 0.01; *** indicates statistical significance at *p* < 0.001.

## Data Availability

The original contributions presented in this study are included in the article. Further inquiries can be directed to the corresponding author.
